# Factors Influencing the Intentions of Patients With Inflammatory Rheumatic Diseases to Use a Digital Human for Medication Information: Qualitative Study

**DOI:** 10.2196/57697

**Published:** 2025-03-13

**Authors:** Lex L Haegens, Victor J B Huiskes, Bart J F van den Bemt, Charlotte L Bekker

**Affiliations:** 1 Department of Research Sint Maartenskliniek Ubbergen The Netherlands; 2 Department of Pharmacy Sint Maartenskliniek Ubbergen The Netherlands; 3 Department of Pharmacy Radboudumc Nijmegen The Netherlands

**Keywords:** digital human, information provision, intention to use, qualitative study, focus groups, drug-related problems, medication safety, safety information, information seeking, Netherlands, Pharmacotherapy, medication, telehealth, communication technologies, medication information, rheumatic diseases, rheumatology

## Abstract

**Background:**

Introduction: Patients with inflammatory rheumatic diseases (IRDs) frequently experience drug-related problems (DRPs). DRPs can have negative health consequences and should be addressed promptly to prevent complications. A digital human, which is an embodied conversational agent, could provide medication-related information in a time- and place-independent manner to support patients in preventing and decreasing DRPs.

**Objective:**

This study aims to identify factors that influence the intention of patients with IRDs to use a digital human to retrieve medication-related information.

**Methods:**

A qualitative study with 3 in-person focus groups was conducted among adult patients diagnosed with an IRD in the Netherlands. The prototype of a digital human is an innovative tool that provides spoken answers to medication-related questions and provides information linked to the topic, such as (instructional) videos, drug leaflets, and other relevant sources. Before the focus group, participants completed a preparatory exercise at home to become familiar with the digital human. A semistructured interview guide based on the Proctor framework for implementation determinants was used to interview participants about the acceptability, adoption, appropriateness, costs, feasibility, fidelity, penetration, and sustainability of the digital human. Focus groups were recorded, transcribed, and analyzed thematically.

**Results:**

The participants included 22 patients, with a median age of 68 (IQR 52-75) years, of whom 64% (n=22) were female. In total, 6 themes describing factors influencing patients’ intention to use a digital human were identified: (1) the degree to which individual needs for medication-related information are met; (2) confidence in one’s ability to use the digital human; (3) the degree to which using the digital human resembles interacting with a human; (4) technical functioning of the digital human; (5) privacy and security; and (6) expected benefit of using the digital human.

**Conclusions:**

The intention of patients with IRDs to use a novel digital human to retrieve medication-related information was influenced by factors related to each patient’s information needs and confidence in their ability to use the digital human, features of the digital human, and the expected benefits of using the digital human. These identified themes should be considered during the further development of the digital human and during implementation to increase intention to use and future adoption. Thereafter, the effect of applying a digital human as an instrument to improve patients’ self-management regarding DRPs could be researched.

## Introduction

Pharmacotherapy is a cornerstone treatment for patients with inflammatory rheumatic diseases (IRDs) [1]. Although disease-modifying antirheumatic drugs (DMARDs) are highly effective in reducing disease activity and increasing daily functioning, patients with IRDs frequently experience drug-related problems (DRPs). Previous research demonstrated that patients with IRDs report on average 5 DRPs within 8 weeks, such as side effects, difficulties with medication management, and information needs [2]. DRPs can potentially lead to negative outcomes of medication use, which, if not addressed promptly, can lead to decreased treatment effects and adverse drug events [3]. Because adverse drug events can increase unplanned hospital admissions and morbidity, and thus health care utilization, DRPs must be addressed quickly to prevent these negative consequences [4-7].

Currently, contact between health care providers (HCPs) and patients is infrequent, with generally up to 4 routine consultations annually. However, DRPs occur throughout the year outside these consultations and could thus be overlooked. Furthermore, patients do not always report DRPs during consultations, and if they do, HCPs do not always act upon them [8]. These factors could delay the identification and resolution of DRPs, potentially putting patients at unnecessary risk. To deliver high-quality chronic disease care according to the Chronic Care Model, patients should be informed and activated, HCPs should be prepared and proactive, and interactions between patients and HCPs should be productive [9].

This study will focus on the informed and activated patient by supporting patient self-management, which can ensure that the limited time HCPs have for contact can be spent on issues that truly require in-person interaction. Furthermore, research shows that patient involvement is essential to identify clinically relevant DRPs promptly [10,11]. This can be achieved by supporting patient self-management by providing medication-related information [12]. It is conceivable that the more patients are informed regarding DRPs, the better they can identify and deal with these DRPs themselves. Thus, supporting patients in becoming more informed could potentially help resolve DRPs on time.

Telehealth, which entails the use of communication technologies to deliver health care at a distance [13], has the potential to support patient self-management through information provision. One such telehealth channel to provide medication-related information is a digital human, which is an embodied conversational agent. Conversational agents are software programs designed to interact with users through natural language dialogue (ie, speech or text), and embodied conversational agents have an additional virtual form represented by an avatar to provide information via face-to-face conversations. Digital humans have successfully been applied in health care, such as for educating patients about care after hospital discharge [14], promoting walking in older patients [15], and facilitating accessibility to web-based health information about breastfeeding [16]. Furthermore, numerous digital humans designed for self-management in chronic diseases have been studied [17].

A digital human offers various advantages for supporting patient self-management, such as time- and place-independent access to information; the ability to process large amounts of information quickly; and intuitive, easily accessible information by mimicking human-to-human interaction [18] (eg, allowing speech as input and output for populations with low literacy levels). It can also provide visual support, such as instructional videos, or nonverbal output like medication leaflets, without requiring involvement from HCPs.

Patients with IRDs reported these advantages of digital humans as influencing their preference for telehealth channels when seeking support for medication use, particularly when offered options with or without HCP contact [19]. Additionally, patients believe telehealth can effectively provide informational support about medication use [12]. Thus, a digital human could support patient self-management by providing easily accessible medication-related information and potentially resolving DRPs more efficiently. However, because using a digital human for providing medication-related information is a novel approach, insights into what factors determine patients’ intention to use a digital human are lacking. Therefore, this study aims to identify factors shaping the intention of patients with IRDs to use a digital human for accessing medication-related information.

## Methods

### Overview

This qualitative study is reported according to the Consolidated Criteria for Reporting Qualitative Research (COREQ) [20].

### Study Design and Setting

An explorative, qualitative study using focus groups was performed. In total, 3 focus groups were held with the aim of reaching data saturation (ie, the point where additional data collection does not produce new relevant themes). For each focus group, between 8 and 12 participants were included. Focus groups were held in the Sint Maartenskliniek Hospital in the Netherlands from February to March 2023.

### Participants

Adult patients (≥18 years of age) were eligible for inclusion if they were prescribed at least 1 DMARD for an IRD from the outpatient pharmacy of the Sint Maartenskliniek Hospital, were proficient in Dutch, and owned an electronic device capable of accessing the digital human (ie, smartphone, tablet, laptop, or desktop) at the time of the study. Participants were purposively sampled based on age, sex, diagnosis, and digital inclination (defined as patients using or not using the web-based personal health record used by the Sint Maartenskliniek Pharmacy). Eligible patients were recruited via email, which contained study information and a form to state availability for the selected dates for focus groups. Written informed consent was obtained in person from participants prior to the focus groups. Incentives for participation were offered in the form of a 20 Euro (US $20.84) gift card, and travel expenses were fully reimbursed.

### Digital Human

**Figure 1:**
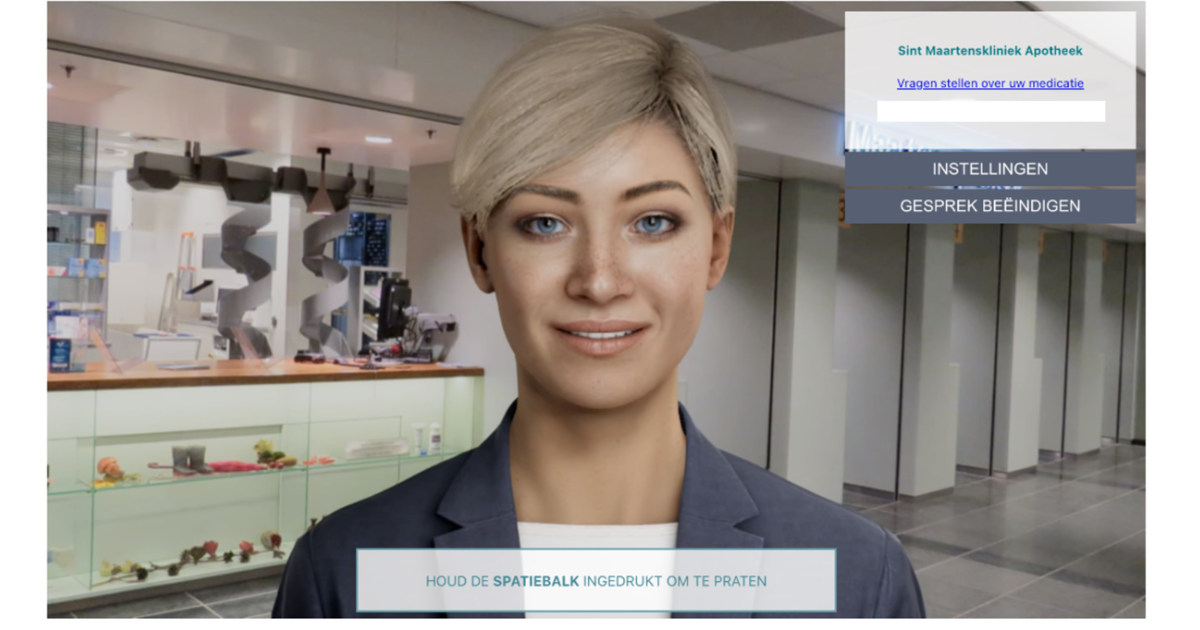
The digital human "Pharmi." Sourced from Pharmi BV.

Pharmi [21], the digital human used during this study, was developed by Pharmi BV and Deloitte in collaboration with the Sint Maartenskliniek Hospital. Pharmi provides spoken answers to drug-related questions and offers additional information, such as (instructional) videos, drug leaflets, and other relevant sources, for approximately 80 different DMARDs. During the study, a prototype of Pharmi was used to provide information and offer predefined answers to common questions, similar to those found on a “Frequently Asked Questions” page. Topics covered included the drug’s mechanism of action, common side effects, instructional videos, storing instructions for antirheumatic drugs, information on specific IRDs, and logistical details such as how to obtain repeat prescriptions (Figure 1).

### Preparation

Participants were asked to complete a preparatory exercise at home prior to the focus group to familiarize themselves with the digital human. This exercise consisted of a set of predetermined frequently asked questions personalized to each participant’s current DMARD and diagnosis (Multimedia Appendix 1). In addition, participants were asked the following questions: (1) “How would you rate the digital human on a scale from 1 to 10?” (2) “What advantages do you see with the digital human? Name a maximum of 2” (3) “What disadvantages do you see with the digital human? Name a maximum of 2.”

### Focus Groups

Focus groups were held at the Sint Maartenskliniek Hospital and lasted 2 hours. Each focus group was led by an experienced moderator (author BJFB or CLB), and an assistant moderator (author LLH) was present to facilitate assignments and take additional field notes. All moderators were researchers at the time of the focus groups. A topic guide was used to standardize and structure each focus group (see Multimedia Appendix 1). This topic guide was based on the implementation outcomes outlined by Proctor et al [22] (ie, acceptability, adoption, appropriateness, costs, feasibility, fidelity, penetration, and sustainability), which are a frequently applied set of outcome measures that describe the concept of implementation outcomes. Service and client outcomes were not included in the topic guide, as these are intended to assess interventions after implementation. All focus groups were audio recorded, and recordings were transcribed verbatim. After 2 focus groups, data saturation was discussed to determine if more than 3 focus groups were necessary.

### Analyses of Transcripts

Transcripts were systematically analyzed using inductive thematic content analysis according to the steps outlined by Braun and Clarke [23] using Atlas.ti 9 (ATLAS.ti Scientific Software Development GmbH) and Microsoft Excel (Microsoft Corp). Transcripts were coded by 3 authors (LLH, VJBH, and CLB). First, open codes were generated for one transcript by LLH and checked by VJBH, after which discrepancies were discussed until consensus was achieved. Second, axial codes were generated by LLH and then discussed by LLH, VJBH, and CLB until consensus was achieved. Third, preliminary themes were constructed by LLH, VJBH, and CLB, after which open and axial coding for the remaining 2 focus groups was performed and themes were revised accordingly. Relevant quotes from participants were selected to support the themes, which were translated into English.

### Ethical Considerations

The Medical Ethics Research Committee of Arnhem-Nijmegen waived official ethical approval (case number 2023-16146) as this study was deemed exempt from the Medical Research Involving Humans Act.

## Results

### Overview

A total of 540 eligible patients were approached for participation, among whom 214 (40%) responded and 31 (6%) agreed to participate. Of these, 9 patients did not participate in a focus group due to sickness (n=4, 44%), sudden unavailability (n=1, 11%) or unspecified reasons (n=4, 44%). Table 1 shows the participants’ characteristics.

**Figure 2:**
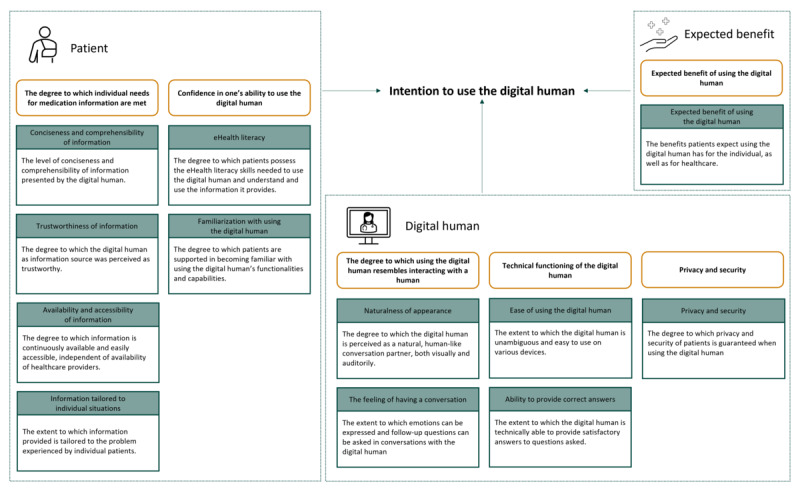
Schematic representation of overarching themes reflecting factors influencing participants' intentions to use a digital human for retrieving medication-related information.

A total of 6 themes reflecting factors influencing participants’ intention to use a digital human to retrieve medication-related information were identified relating to the patient, digital human, and the expected benefit from using the digital human (Figure 2).

**Table 1:** Characteristics of the study population (N=22).

Age (years), median (IQR)			68 (52-75)
Female sex, n (%)			14 (64)
**Diagnosis, n (%)**			
	Rheumatoid arthritis	14 (64)	
	Psoriatic arthritis	6 (27)	
	Other	2 (9)	
Digital inclination (yes), n (%)			11 (50)

### Theme 1: Degree to Which Individual Needs for Medication-Related Information Are Met

#### Explanation

The intention to use a digital human to retrieve medication-related information was dependent on patients’ individual information needs. These needs varied between participants and related to different aspects of the information, and in some cases, the problem experienced.

#### Conciseness and Comprehensibility of Information

Most participants preferred the concise information presented by the digital human compared with the relatively abundant information found in medication information leaflets, general brochures about medical treatment, and other web-based information sources. On the other hand, some participants felt that the digital human presented limited information compared with other medication information sources.

For some people, listing five main side effects is sufficient, but I also know that some people do not dare to take the medication because it has so many side effects.

Female, 75 years

Participants reported comprehensibility of information as a factor that influenced their intention to use the digital human. Although some participants found the information presented by the digital human rather vague, others appreciated the clear information it provided. The degree to which the information provided by the digital human was experienced as comprehensible varied. Additionally, the digital human did not verify that the patient understood the information provided.

#### Trustworthiness of Information

The participants highlighted the importance of the trustworthiness of information provided by the digital human. They perceived information provided by the digital human as trustworthy, particularly when compared to web-based sources, as the digital human was deployed by the hospital. Participants suggested that trustworthiness would increase if they were informed about the availability of the digital human by reliable authorities, such as patient associations or HCPs from the hospital where they were treated. Furthermore, participants trusted that the digital human would provide the most up-to-date information available.

If you look at the digital human you get the feeling the information is provided by the pharmacist, and if you just search on Google, you get the feeling it is provided by someone random. So, I see that as an advantage, I would have more trust in that [the digital human].

Female, 78 years

#### Availability and Accessibility of Information

The participants expressed a desire for continuously available information. They sometimes felt that they had to wait for the next HCP consultation to retrieve information and questioned this relatively low accessibility of information. They mentioned that the digital human fulfilled the need for easily accessible information by being available 24/7 without having to wait for an HCP to become available.

The benefit of this [the digital human] is that I do not have to wait until I see my treating physician again. I do not have to travel to the hospital to get answers.

Male, 76 years

The digital human was regarded as a fast information source that could directly answer questions, whereas, with other information sources, patients had to search for relevant information more intensively.

#### Information Tailored to Individual Situations

Another factor influencing patients’ intentions was to what extent the information was tailored to their concerns. Participants intended to use the digital human for general questions, such as medication side effects or potential medication interactions. For more specific, personal questions, such as regarding the choice of treatment, participants’ intention to use the digital human was lower. The main reason was that the digital human provided predefined answers to predefined questions and could not generate tailored information.

Well, it is all programmed. If you have really detailed questions, I wouldn’t even bother trying, I think.

Female, 67 years

### Theme 2: Confidence in One’s Ability to Use the Digital Human

#### Explanation

The participants found it important to feel confident in using the digital human, as this would increase their chances of using it more often.

#### eHealth Literacy

The participants felt that adequate eHealth literacy was needed for using the digital human, including digital skills and health literacy. Regarding digital skills, patients indicated that proficiency in using an electronic device on which the digital human was available was necessary. Some participants believed that older people could be less digitally skilled and thought the digital human was less suitable for this age group.

Of course, there are also many people, especially in the older population, who experience difficulties with all digital tools.

Male, 77 years

Furthermore, participants felt that health literacy was important for adequately using the digital human. It would enable patients to formulate and articulate questions clearly and understand and apply the health-related information provided. Since the digital human focuses on verbal communication, participants believed it could be especially beneficial for individuals with low literacy (ie, those who struggle with reading and writing), enabling them to ask questions verbally and receive spoken responses.

#### Familiarization With Using the Digital Human

Participants noted that familiarity with using the digital human would increase their intention to use it. They suggested that HCPs provide clear instructions and support during initial use. Additionally, participants emphasized the importance of having sufficient time to explore and understand the digital human’s functionalities and capabilities, as this would increase their confidence and intention to use it. However, they also highlighted that the quality of their early experiences (whether positive or negative) would influence their use intention.

…The fact that you get used to talking with someone, communicating with someone who does not exist...

Male, 76 years

### Theme 3: Degree to Which Using the Digital Human Resembles Interacting With a Human

#### Explanation

The extent to which interaction with the digital human resembled human-to-human interaction when retrieving medication-related information influenced participants’ intention to use it. Some participants believed that a human-like interaction would positively influence their intention to use the digital human, while others indicated that this did not impact their intention.

#### Naturalness of Appearance

The perceived naturalness and human-like qualities of the digital human influenced the participants’ intention to use it. They felt that the digital human appeared artificial and lacked sufficient human traits. Specifically, they felt that the digital human did not speak like a real person, as it misplaced emphases in sentences, lacked proper pronunciation, and had a tinny tone, leading to nonfluent communication. While the presence of a digital human image increased some participants’ attention to the provided answers, others found the unnatural facial expressions and constant movement of the image distracting, negatively influencing their use intention.

#### The Feeling of Having a Conversation

Participants found interactions with the digital human more impersonal, citing the inability to express emotions compared to conversations with an HCP.

When interacting with the digital human, I don't feel that I can genuinely express certain emotions in that interaction.

Male, 76 years

Furthermore, participants viewed the lack of follow-up question capabilities negatively. They noted that the digital human did not allow users to ask follow-up questions after receiving an answer, nor did it initiate follow-up questions itself, further diminishing the sense of a meaningful dialogue with the digital human.

The digital human, who, by the way, looks very pleasant, only provides preprogrammed responses. So, there is no dialogue where you can ask a question yourself.

Male, 77 years

### Theme 4: Technical Functionalities of the Digital Human

#### Explanation

The technical performance of the digital human influenced the participants’ intention to use it. Participants expressed frustration when retrieving information was difficult, which negatively influenced their intention to use the digital human.

#### Ease of Using the Digital Human

Participants emphasized that using the digital human should feel straightforward and easy. While some found it easy to use, others did not due to the prototype’s technical flaws. Several issues were reported regarding the synchronicity between the digital human’s image and sound. This was especially problematic for patients who rely on lip-reading.

What I found very difficult, as I have a hearing impairment, I have a hearing aid and heavily rely on lip reading, the digital human does not synchronize. I think... Well, that's a turn-off for me.

Female, 50 years

Additionally, participants found using the digital human on a mobile phone more challenging compared to a laptop or computer. They stressed that the digital human should function smoothly on various types of devices and be easy to read.

Participants had different opinions on the importance of a safe login procedure. Most felt that requiring a login was too cumbersome, while others valued the security, especially if a login could lead to more tailored answers based on the patient’s current medication extracted from a personal health record.

#### Ability to Provide Correct Answers

Many participants experienced difficulties receiving satisfactory answers, mainly due to the digital human not understanding their questions. In several cases, participants did not receive an answer at all, while in other cases, the digital human answered a different question than the one asked. Participants reported that they became impatient and frustrated when having to repeat the question, negatively influencing their intention to use the digital human. Moreover, participants noted that some medication names are difficult to pronounce, which can vary across individuals. They suggested improving speech recognition so the digital human could better handle different pronunciations and accents.

If “Have you not heard it correctly,” then you have to repeat [your question]. You know, you become impatient.

Female, 50 years

It mainly has to do with how you pronounce the medication, if she [the digital human] understands it. And I also think it depends on the dialect. My wife tried it as well, there she [the digital human] worked and for me, it did not.

Male, 79 years

### Theme 5: Privacy and Security

Participants emphasized the importance of privacy and security in their intention to use the digital human. Regarding security, they expressed worries about the possibility of microphones being tapped and the risks associated with cookies from other websites, which could negatively influence their intention to use the digital human. Some participants felt privacy was guaranteed, as questions could be asked anonymously.

However, others had concerns about privacy when using the digital human in public spaces, as verbal questions and answers could be heard by others.

I thought that with anonymity, perhaps some people might feel embarrassed to ask questions because they don't know (…)which they think everyone should know. That's why anonymity may be nice.

Female, 75 years

### Theme 6: Expected Benefits of Using the Digital Human

The anticipated benefits of using the digital human, both at the individual level and societal level, influenced participants’ intentions to use it. On an individual level, the digital human was perceived as a tool to enhance self-management by giving patients more control over their own care.

I think it is beneficial for health care providers, the better informed patients are about their medication, the better they are doing. As I just said, with medication adherence, but also with trust patients have in their medication. I think that is very important.

Male, 77 years

The participants viewed the digital human as a way to increase their knowledge about medication and thus their involvement in their treatment. They felt that the digital human enabled them to address issues independently without having to reach out to HCPs, thereby avoiding additional burdens on HCPs.

As has been said before, you don't have to bother anyone, you can look it up yourself.

Male, 79 years

Furthermore, participants believed that using the digital human could improve their digital skills and preparedness for the digitization of health care.

## Discussion

### Principal Findings

This qualitative study showed that the intention of patients with IRDs to use a digital human for retrieving medication-related information was influenced by several factors. These factors included individual patients’ information needs and their confidence in using the digital human; the digital human’s features, including human-like interaction and technical functionalities; and the expected benefits of using it.

Over the past decade, digital humans have been developed for various chronic conditions to facilitate screening, diagnosis, and education [17]. However, using a digital human specifically for retrieving medication-related information in rheumatic diseases is a novel approach. As a result, direct comparisons with earlier studies investigating digital humans in the same context are difficult.

In this study, the intention to use the digital human largely depended on patients’ individual information needs, including their need for concise, trustworthy, accessible, and tailored information. These factors have also been described in other studies regarding the information needs of patients with rheumatic diseases [12,24,25]. Additionally, patients’ confidence in their ability to use the digital human and their eHealth literacy skills influenced their use intentions. This aligns with previous research showing that health literacy is associated with the ability to retrieve online health information [26,27] and evaluate its quality [28].

Another theme influencing intention was the ease of use of the digital human. This relation is widely documented in frameworks such as the Technology Acceptance Model, which describes “perceived ease of use” as a factor that indirectly influences the intention to use [29]. Given that the digital human used in this study was a prototype, participants’ remarks regarding ease of use should be considered during further development to maximize future intentions to use and thus adoption.

Additionally, participants emphasized the importance of the digital human’s technical functionalities, particularly its ability to provide correct answers. A mixed methods study on the use of digital humans for patients with dementia similarly found that technical problems and issues with speech recognition negatively impacted patient’s intention to use [30]. In this study, participants also indicated that a negative first experience with the digital human (ie, an experience in which a correct answer was not provided by the digital human) could deter future use intention. Therefore, ensuring an optimal first experience is critical for successful implementation and adoption by patients.

Research on digital humans applied in different contexts suggests that design features, such as facial expressions and tone of voice, influence use intentions [31]. For example, a previous study on a digital human for type 2 diabetes self-management found that its acceptability partly depended on the congruence between its verbal and nonverbal communication [32]. Similarly, in this study, participants indicated that the degree to which the digital human resembled a natural, human-like conversation partner influenced their intention to use it.

Various communication and information channels are available in health care, including frequently asked questions, phone or video calls, and 2-way text messaging with HCPs. These channels vary in interaction levels, user experiences, accessibility, and monetary or personnel costs associated with using the channel. It is conceivable that the digital human is not always the most suitable channel in every situation and for every individual (eg, a patient can experience multiple problems according to which information should be tailored). However, offering the digital human alongside existing channels allows patients to choose the option that best matches their needs and preferences. This could increase the actual usage of such channels to retrieve medication information, leading to patients gaining more medication knowledge, which is recognized as an important element of patients’ self-management [33].

### Strengths and Limitations

A notable strength of this study is its theory-based approach to constructing the topic guide, which was informed by the Proctor framework for successful implementation [22]. The factors identified in this study are valuable for integrating the digital human into routine care. For example, integrating the digital human into digital patient health records could enhance the trustworthiness and accessibility of medication information. These factors are also relevant for further development of the digital human to increase future adoption. For example, speech recognition could be improved to increase the digital human’s ability to provide correct answers. This is especially valuable because the uptake of telehealth is low among patients with IRDs despite their willingness to use it [34].

A few limitations should be acknowledged. First, the digital human used in this study was a prototype and had several technical flaws, such as suboptimal processing of participants’ questions participants and difficulties in controlling the digital human. These issues may have influenced the range and type of technical factors participants identified as impacting their intention to use the digital human.

Second, the participant group was slightly older than the nonparticipant group. The nonparticipants’ median age was 59 (IQR 49-68) years. This might be explained by the fact that 2 out of 3 focus groups focus groups were held face-to-face during office hours, potentially making it easier for older adults with more spare time to attend. Additionally, this study had a low participation rate (6%). Most patients did not respond to the online invitation, and those who declined to participate were not obliged to provide reasons, leaving the reasons for nonparticipation unknown. Third, the participants were recruited from a single, specialized rheumatology clinic and might differ from the general IRD population in in terms of their experiences with the level of quality of care and medication information provided. This may limit the generalizability of the study’s findings.

### Conclusion

This qualitative study highlights that the intention of patients with IRDs to use a novel digital human for medication-related information is influenced by their individual information needs, confidence in their ability to use the digital human, its features, and the expected benefits of using the digital human. These themes should guide the development and implementation of a digital human to maximize patients’ intention to use and adoption. Future research should examine the effect of applying the digital human as a tool to improve patients’ self-management of DRPs.

## Appendix

Multimedia Appendix 1 Questions for focus group participants to prepare for focus groups and topic guide used during focus groups.

## Abbreviations

COREQ: Consolidated Criteria for Reporting Qualitative Research
DMARD: disease-modifying antirheumatic drug
DRP: drug-related problem
HCP: health care provider
IRD: inflammatory rheumatic disease
