# Supplementary material for: Factors Influencing the Intentions of Patients With Inflammatory Rheumatic Diseases to Use a Digital Human for Medication Information: Qualitative Study
Source: J Med Internet Res. 2025 Mar 13;27:e57697. doi: 10.2196/57697 (PMC11950694; doi:10.2196/57697)
Supplement: Multimedia Appendix 1 [file jmir_v27i1e57697_app1.docx]

**Manuscript ‘**Factors influencing the intention of patients with inflammatory rheumatic diseases to use a digital human to retrieve medication-related information: a qualitative study’

Supplementary materials 1

**Preparatory questions**

1. How would you rate the digital human after completing the preparatory exercises on a scale from 1 to 10?
2. What are the digital human’s most important advantages?
3. What are the digital human’s most important disadvantages?

**Focus group topic guide**

1. Discuss preparatory questions
   1. How would you rate the digital human after completing the preparatory exercises on a scale from 1 to 10?
   2. What are the digital human’s most important advantages?
   3. What are the digital human’s most important disadvantages?
2. Appropriateness and acceptability of the digital human
   1. To what degree do you think the digital human is an appropriate way to resolve medication-related questions?
   2. To what degree would you use the digital human to resolve medication-related questions?
3. Ease of use of the digital human
   1. To what degree do you think you are able to successfully use the digital human to resolve medication-related questions?
   2. When comparing the digital human to other ways one can resolve medication-related questions, what are reasons you would or would not use the digital human to resolve medication-related questions?
   3. How can we facilitate patients to use the digital human for resolving medication-related questions, and ensure future use?
4. Possible value of the digital human
   1. What added benefit do you believe the digital human can have for healthcare?
